# Morpho-functional changes of cardiac telocytes in isolated atrial amyloidosis in patients with atrial fibrillation

**DOI:** 10.1038/s41598-021-82554-0

**Published:** 2021-02-11

**Authors:** Tatyana V. Sukhacheva, Natalia V. Nizyaeva, Maria V. Samsonova, Andrey L. Cherniaev, Artem A. Burov, Mariia V. Iurova, Aleksandr I. Shchegolev, Roman A. Serov, Gennady T. Sukhikh

**Affiliations:** 1 A.N. Bakulev National Medical Research Center of Cardiovascular Surgery, The Ministry of Health of Russian Federation, Moscow, Russia; 2 National Medical Research Center for Obstetrics, Gynecology, and Perinatology Named After Academician V.I. Kulakov of the Ministry of Healthcare of the Russian Federation, Moscow, Russia 117997; 3 Pulmonology Scientific Research Institute under Federal Medical and Biological Agency of Russian Federation, Moscow, 115682 Russia; 4 First Moscow State Medical University Named After I.M. Sechenov, Moscow, Russia

**Keywords:** Atrial fibrillation, Heart stem cells, Mechanisms of disease

## Abstract

Telocytes are interstitial cells with long, thin processes by which they contact each other and form a network in the interstitium. Myocardial remodeling of adult patients with different forms of atrial fibrillation (AF) occurs with an increase in fibrosis, age-related isolated atrial amyloidosis (IAA), cardiomyocyte hypertrophy and myolysis. This study aimed to determine the ultrastructural and immunohistochemical features of cardiac telocytes in patients with AF and AF + IAA. IAA associated with accumulation of atrial natriuretic factor was detected in 4.3–25% biopsies of left (LAA) and 21.7–41.7% of right (RAA) atrial appendage myocardium. Telocytes were identified at ultrastructural level more often in AF + IAA, than in AF group and correlated with AF duration and mitral valve regurgitation. Telocytes had ultrastructural signs of synthetic, proliferative, and phagocytic activity. Telocytes corresponded to CD117^+^, vimentin^+^, CD34^+^, CD44^+^, CD68^+^, CD16^+^, S100^-^, CD105^-^ immunophenotype. No significant differences in telocytes morphology and immunophenotype were found in patients with various forms of AF. CD68-positive cells were detected more often in AF + IAA than AF group. We assume that in aged AF + IAA patients remodeling of atrial myocardium provoked transformation of telocytes into “transitional forms” combining the morphological and immunohistochemical features with signs of fibroblast-, histiocyte- and endotheliocyte-like cells.

## Introduction

Telocytes (TCs)—is recently discovered interstitial cells described in the myocardium^1–13^, as well as in various organs of human and other mammals^14–29^. TCs morphology is similar to Cajal pacemaker cells of the gastrointestinal tract. TCs are usually spindle-shaped and differ from all known types of connective tissue cells by the presence of thin processes (telopodes, Tps), the length of which reaches 100 μm. In recent years, the possible role of cardiac TCs has been actively discussed. The processes of the TCs “accompany” all components of the interstitium—cardiomyocytes, blood vessels, nerve fibers. TCs contact with each other and with other interstitial cells due to nexuses, point contacts, or nanocontacts^6,30^. They are performed a structural and a coordination bond between all cell types^2,6,9,13,31,32^. TCs number in atrial myocardium is higher than in ventricles, and in epicardium is higher than in endocardium^33^. It is assumed that in atrial myocardium and in myocardial sleeves of human pulmonary veins, TCs have pacemaker activity^1,2^. They probably act as alternative generators of electrical impulses. TCs have direct nanocontacts with endotheliocytes and thus provide structural and functional support for endothelial cells, create a microenvironment, and participate in angiogenesis^6,34–36^. Moreover, TCs organize the architectonics of myocardial tissue and determine the localization and differentiation of stem cells in the processes of growth and regeneration of cardiac muscle tissue^1,2,6,7,31,33–35,37,38^, similar to those occurring in skeletal muscle during the interaction of TCs with resident satellite cells^29^.

A change in the structure of the interstitium in atrial myocardium is one of the reasons for the development and maintenance of AF. The major morphological features of atrial myocardial remodeling in patients with AF are increased fibrosis, lipomatosis, and development of isolated atrial amyloidosis (IAA), which correlates with the age of patients with AF^39–44^. Amyloidosis is a process associated with impaired protein metabolism and is accompanied by the formation and deposition in tissues of a specific protein-polysaccharide complex—amyloid. Amyloidosis is usually a systemic disorder with multiple organ damage affected. Nevertheless, the myocardium of patients with AF is characterized by a heart-specific type of amyloidosis—IAA. According to some authors, IAA is associated with the deposition of a fibrillar form of atrial natriuretic peptide (ANP) in atrial myocardium^41,44–50^. Normally, ANP is predominantly synthesized by atrial myocardium, found in electron-dense atrial granules in cardiomyocytes, and released in response to atrial wall extension. ANP activates transmembrane receptors in target organs, has a natriuretic, diuretic, and vasodilatory effect, and inhibits the renin–angiotensin–aldosterone system. Plasma levels of ANP are increased in patients with heart failure caused by cardiac valve pathology, coronary heart disease, congenital heart defects, dilated and hypertrophic cardiomyopathies, and AF compared with control patients^51–55^. An analysis of the literature data suggests a relationship between AF and IAA^41–43,48,56–58^. Accumulations of amyloid fibrils in atrial myocardium of patients with AF surround and isolate cardiomyocytes and all interstitial components, interfering with contacts between cells, leading to their separation and disruption of electrical conductivity. They are a factor that provokes or supports AF.

The morphological features of TCs at the ultrastructural level in pathologically altered myocardium is still unclear yet. In humans and mammals with AF, TCs have been detected in atrial myocardium^43,59^ and myocardial sleeves of human pulmonary veins^13,17,31^. The authors have noticed heterogeneity in the distribution of TCs—they were not found in all patients and not in all samples^17^. In myocardium with significant sclerosis, a decrease in the number of TCs have been found^27,59,60^. TCs disappeared in the myocardium after a heart attack, but after a while, their number was increased in the peri-infarction zone, which confirmed the participation of TCs in tissue regeneration^7,33,34,61^.

The present study was focused on the analysis of the morphology of the left (LAA) and right (RAA) atrial appendages myocardium in patients with different forms of AF (paroxysmal (PAF), persistent (PsAF) and long-standing persistent (LSAF) with special emphasis on the study of ultrastructural features of interstitial cells—TCs in two groups of patients: with AF and AF + IAA. We expected to define TCs function and identify these cells using immunohistochemical markers. The data obtained were compared with the morphological parameters of the myocardium and the clinical parameters of patients. We suggested that in patients with different forms of AF hemodynamic overload provokes various types of pathological remodeling of atrial appendage myocardium and expected to make a comparative analysis of the increase in fibrosis, lipomatosis, IAA and hypertrophy of cardiomyocytes. Moreover, we hypothesized that interstitial tissue reorganization can initiate the transformation of TCs with the appearance of “transitional forms” combining the morphological and immunohistochemical characteristics of TCs with signs of fibroblast-like cells, histiocyte-like cells, and endotheliocytes.

## Results

### Light microscopy

**Figure 1:**
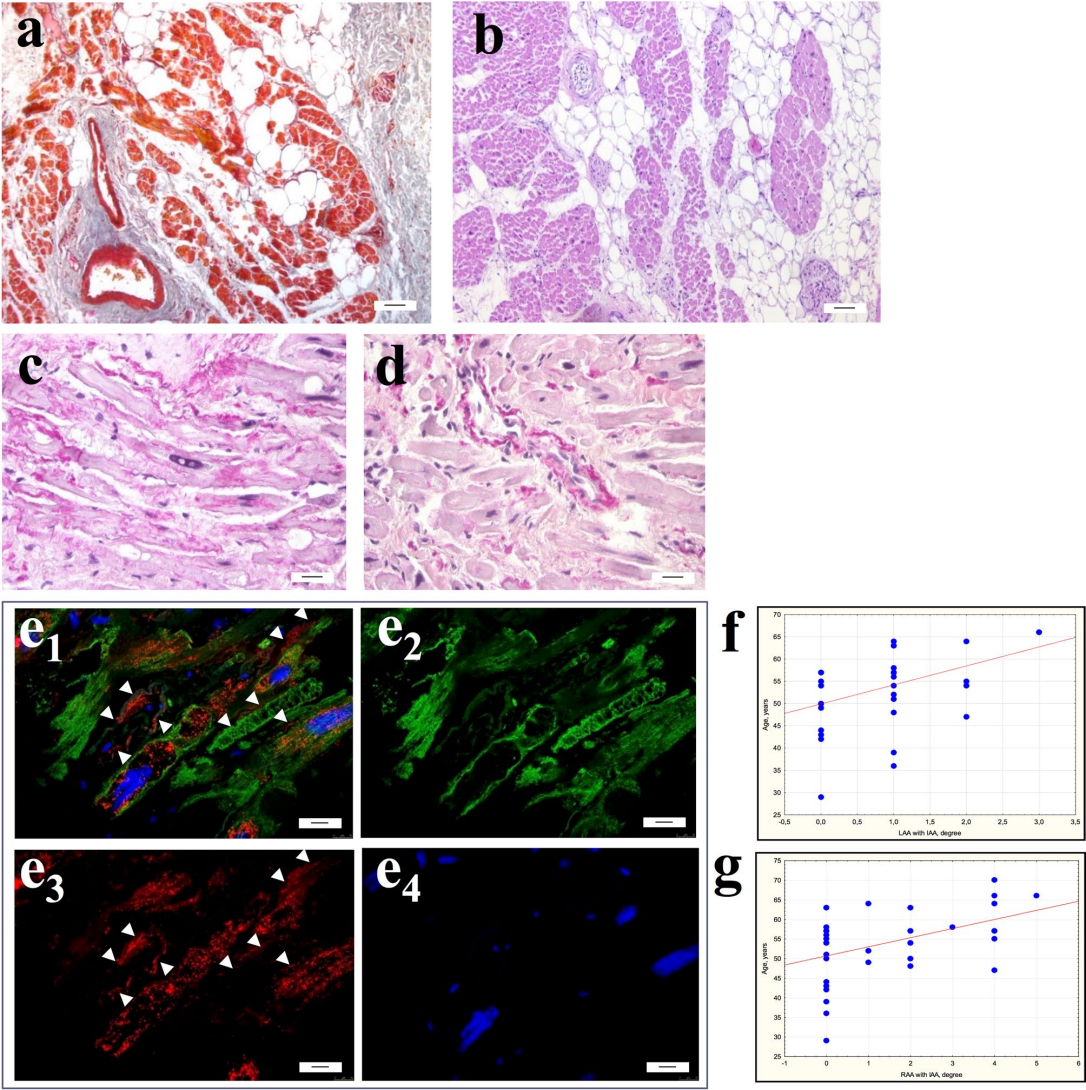
Interstitial area of atrial appendage myocardium of patients with AF. (**a**,**b**) Significant fibrosis and lipomatosis in the interstitium of atrial appendage myocardium. (**a**) Masson’s trichrome stain. (**b**) Hematoxylin and eosin. bar 7 µm. (**c–e**) Isolated atrial amyloidosis in atrial appendage myocardium. Amyloid deposition in the interstitial area on the surface of cardiomyocytes (**c**) and the wall of the intramural vessel (**d**). (**c**,**d**) Sirius red stain. bar 30 µm. e_1-4_—ANP^+^ fibrillar material in the interstitium on the surface of cardiomyocytes sarcolemma and in the walls of blood vessels (arrows). ANP^+^ atrial granules in the sarcoplasm of cardiomyocytes. e_1_—ANP/Desmin/DAPI immunohistochemistry. e_2_—Desmin-positive fibrils in the sarcoplasm of cardiomyocytes (Alexa 488). e_3_—Detection of ANP-positive atrial granules in the sarcoplasm of cardiomyocytes and ANP-positive fibrils in the interstitium (arrows) (Alexa 546). e_4_—DAPI-stained nuclei. Immunoconfocal laser microscopy. bar 10 µm. **(f**,**g**) Correlation of the amyloid distribution in the LAA (**f**) and RAA (**g**) myocardium with the age of patients with AF.

In light microscopy, the interstitium of atrial appendage myocardium of patients with AF was characterized by significant sclerotic changes and lipomatosis (Fig. 1a,b). In patients with different forms of AF, the percentage of fibrosis did not differ and averaged 45.8–47.0% in the LAA myocardium and 44.4–50.3% in the RAA myocardium. The maximum fibrosis reached 61.8–74.7% (Table 1). Cardiomyocytes were usually hypertrophied: their diameters were on average 14.9–16.3 μm (LAA) and 15.4–15.9 μm (RAA) and did not differ significantly in patients of various groups with AF (Table 1). Diameters of cardiomyocytes of patients suffering from PAF and PsAF correlated with an increase in the length of these cells (PAF: RAA; PsAF: LAA, RAA; *p* < 0.05). In patients with LSAF the length of cardiomyocytes significantly exceeded that in patients with PAF (LAA and RAA, *p* < 0.05) and PsAF (RAA, *p* < 0.05) (Table 1) and correlated with the duration of AF (LSAF r = 0.59; *p* = 0.0096). In the LAA myocardium of 25–50% of patients and in the RAA myocardium of 33–45% of patients with different forms of AF, cardiomyocytes with partial loss of myofibrils (myolysis) were detected. The presence of myofibril free zones negatively affected the contractile activity of cardiomyocytes, since a large proportion of the sarcoplasm of these cells did not contain myofibrils and was filled with glycogen granules and mitochondria. Nevertheless, the proportion of cardiomyocytes with myolysis did not differ between patients of various groups with AF (Table 1).

**Table 1:** Morphological characteristics of atrial appendages myocardium of patients with different forms of AF (PAF, PsAF, LSAF) and two groups with AF and AF + IAA.

Morphological parameters	LAA/RAA	Patients with different forms of AF			Patients with IAA or AF + IAA	
		PAF (n = 12)	PsAF (n = 20)	LSAF (n = 23)	AF (n = 37)	AF + IAA (n = 18)
Fibrosis Me (min–max), %	LAA	47.0 (24.7–61.8)	45.8 (36.7–63.4)	46.9 (3.17–60.1)	46.6 (3.2–63.4)	44.0 (24.7–61.8)
	RAA	46.9 (21.5–54.1)	44.4 (19.3–61.6)	50.3 (3.22–74.7)	45.7 (21.5–74.7)	44.6 (19.3–52.6)
IAA, % of patients	LAA	16.7	25	4.3	-	100
	RAA	41.7	30	21.7	-	100
Cardiomyocyte diameter, mean ± SD, µ	LAA	14.9 ± 1.5	16.0 ± 2.8	16.3 ± 2.4	15.5 ± 2.2	16.6 ± 2.8
	RAA	15.4 ± 2.0	15.7 ± 3.2	15.9 ± 2.4	15.2 ± 2.4	16.5 ± 2.7
Cardiomyocyte length, mean ± SD, µ	LAA	59.7 ± 13.0*	67.1 ± 8.9*	73.1 ± 8.2	68.4 ± 10.3	66.4 ± 12.2
	RAA	68.5 ± 3.6	68.3 ± 9.8*	74.4 ± 13.5	69.1 ± 11.1	73.6 ± 10.3
Cardiomyocytes with myolysis (10–50% and more 50% of cell volume), % of patients	LAA	25	50	26.1	35.1	33.3
	RAA	33.3	45	43.5	40.5	44.4

*Comparison with LSAF, Mann–Whitney U Test *p* < 0.05.

In patients with AF accumulations of Sirius red-stained amyloid deposits were detected in the interstitial area on the surface of cardiomyocytes sarcolemma and at the walls of the intramural vessels of atrial appendage myocardium (Fig. 1c,d). An immunohistochemical study using specific antibodies confirmed the ANP origin of fibrillar amyloid protein, which was typical for IAA (Fig. 1e). 4.3–25% of patients with different forms of AF were found to have significant amyloid deposition in LAA myocardium and 21.7–41.7% of patients—in RAA myocardium, although these values did not differ significantly (Table 1). We found that IAA was an age-related factor of the LAA (r = 0.40; *p* = 0.015) and the RAA (r = 0.42; *p* = 0.01) myocardial remodeling (Fig. 1f,g). IAA was more common in the LAA myocardium in patients with PsAF (25%) and in the RAA myocardium in patients with PAF (41.7%). IAA was more pronounced in women than in men (PAF: r = 0.62; *p* = 0.04; PsAF: r = 0.54; *p* = 0.03; LSAF: r = 0.47; *p* = 0.04).

The above morphological characteristics of the myocardial interstitium and cardiomyocytes did not differ between AF groups, with the exception of the length of the cardiomyocytes (Table 1). At the same time, correlation analysis made it possible to identify certain patterns of changes in morphological parameters within each of the groups. In patients with PAF, an increase in the diameters of LAA cardiomyocytes correlated inversely with the size of the left atrium (r = − 0.63; *p* = 0.038) and positively correlated with the presence of IAA (r = 0.67; *p* = 0.02). Areas of sarcoplasm without myofibrils were mainly detected in LAA cardiomyocytes of small diameter (r = − 0.77; *p* = 0.005) in patients with progressive dilatation of the left atrium (r = 0.69; *p* = 0.02), mitral valve regurgitation (r = 0.68; *p* = 0.02) and a shorter AF duration (r = − 0.65; *p* = 0.03). Furthermore, in patients with PAF and IAA, cardiomyocytes with myolysis were less common (LAA: r = − 0.73; *p* = 0.02; RAA: r = − 0.84; *p* = 0.009), in turn, IAA was more pronounced in patients with a long duration of AF (LAA: r = 0.79; *p* = 0.011). In patients with PsAF, an increase in left atrial volume positively correlated with IAA (r = 0.72; *p* = 0.004), but inversely with LAA myocardial fibrosis (r = − 0.68; *p* = 0.03) and IAA inversely correlated with fibrosis (LAA: r = − 0.60; *p* = 0.049). LAA cardiomyocytes with myolysis were detected in PsAF patients with long AF duration (r = − 0.59; *p* = 0.009). In LSAF patients, an increase in the diameters of LAA cardiomyocytes positively correlated with an increase in the left atrium size (r = 0.48; *p* = 0.025), dilatation of the fibrous ring of the mitral valve (r = 0.77; *p* = 0.0002), and an increase in RAA cardiomyocytes diameters positively correlated with dilatation of the fibrous ring of the tricuspid valve (r = 0.44; *p* = 0.045). In contrast to the data for the PAF group, in patients with LSAF, an increase in the diameter of RAA cardiomyocytes was accompanied by the loss of myofibrils in them (r = 0.65; *p* = 0.0014), moreover, cardiomyocytes with myolysis were more common in the myocardium with IAA (r = 0.56; *p* = 0.015).

When dividing patients with AF into two groups according to the presence of IAA. the above indicated morphometric measurements of the proportion of fibrosis, the diameters and the lengths of cardiomyocytes, proportions of patients with myolysis in cardiomyocytes did not differ significantly in LAA and RAA myocardium of patients with AF and AF + IAA (Table 1).

### Transmission electron microscopy (TEM)

**Figure 2:**
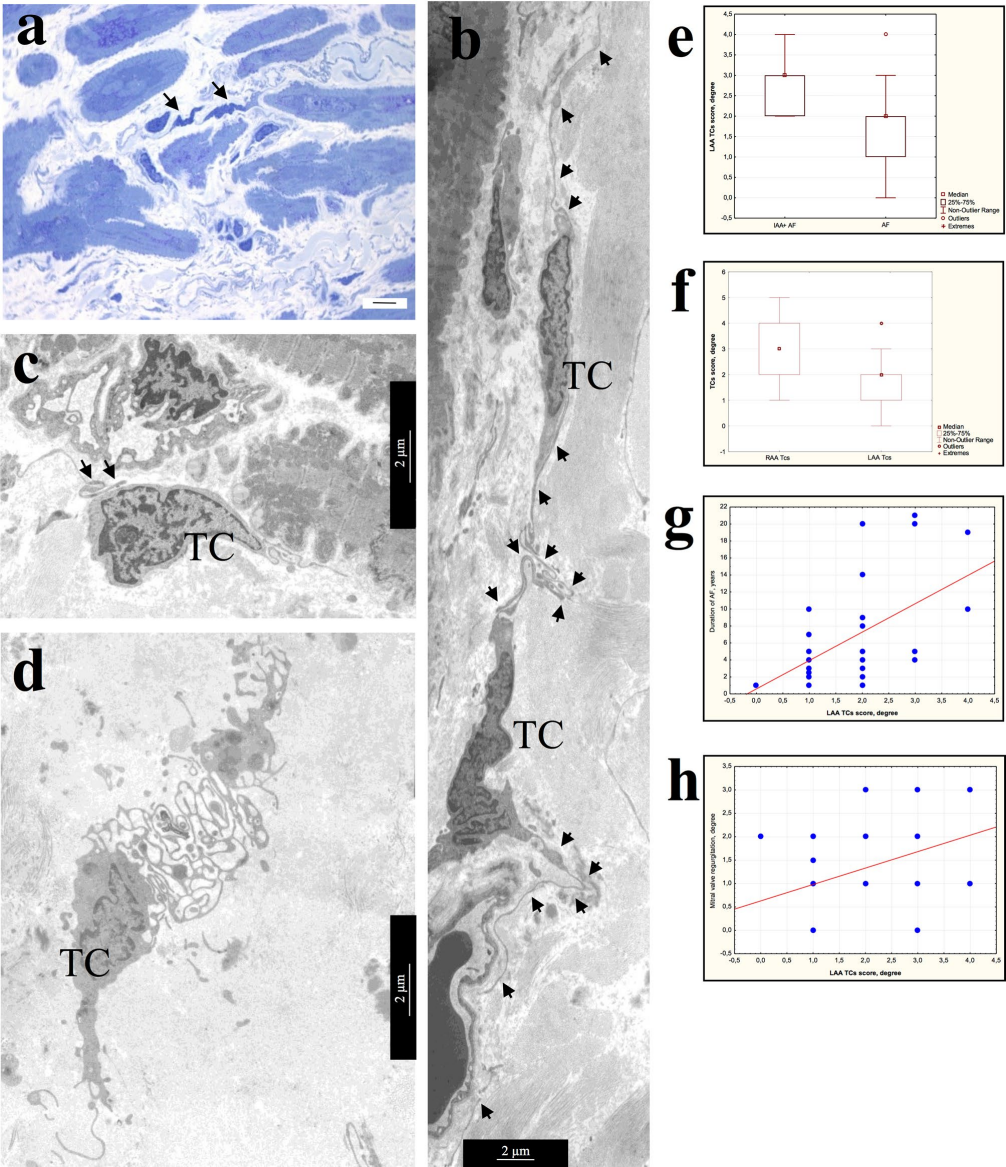
Morphology of TCs in atrial appendage myocardium of patients with AF. (**a**) Comparative morphology of TCs (arrows) and cardiomyocytes. Semi-thin sections. PAS reaction with methylene blue staining. bar 10 µm. (**b)** Spindle-shaped and star-shaped TCs with 2–3 processes (arrows). (**c**) Contact of the TC process (arrows) with the “body” of another TC. (**b**,**c**) bar 2 µm. (**d**) TC with numerous branching processes that form a labyrinthic system. b-d Transmission electron microscopy (TEM). (**b**,**c**) bar 2 µm. TC—telocyte. (**e–h**) Semi-quantitative assessment of presence TCs in atrial appendage myocardium. (**e**) Comparison of TCs presence in LAA myocardium of AF + IAA patients and AF patients (Mann–Whitney test, *p* < 0.05). (**f**) The ratio of TCs score in LAA and RAA myocardium (Mann–Whitney test, *p* < 0.05). (**g**,**h**) Positive correlation of TCs score in LAA myocardium with the duration of AF (**g**, r = 0.50; *p* = 0.0035), the degree of mitral valve regurgitation (**h**, r = 0.37; *p* = 0.033).

Light microscopy, using semi-thin sections and TEM investigations of atrial appendage myocardial samples allowed us to identify TCs in the myocardium of patients of all AF groups by their morphology and location in the interstitial area (Fig. 2a). It was important to estimate the size and shape of these cells and the presence of thin, long processes—Tps. Some areas of Tps have local extensions (podoms), that contain mitochondria, rough endoplasmic reticulum (rER), and vesicles. The number of TCs in the myocardium of different patients varied significantly, but did not differ in groups of patients with various forms of AF. In some samples, single TCs and fragments of their processes were found; in other cases, Tps of numerous TCs formed a dense three-dimensional network in the interstitial area. TCs were small cells, whose dimensions were significantly smaller than the adjacent cardiomyocytes. Thus, an average TCs diameter was 2.1 ± 0.6 μm (0.8–3.4 μm), and an average diameter of Tps was 0.2 ± 0.15 μm (0.03–1.92 μm), while an average diameter of atrial appendage cardiomyocytes exceeded 15–16 μm on average (Fig. 2a, Table 1). As a rule, TCs were spindle-shaped or star-shaped with 2–5 long Tps, by which they contacted each other. The spindle-shaped TCs, as a rule, had two Tps, star-shaped TCs—three or more Tps (Fig. 2b,c). Sometimes TCs with unusual branching processes formed a labyrinthic system in the interstitium, the function of which was not clear (Fig. 2d). The TCs shape, size, and number of processes did not differ among patients with various forms of AF, but were associated with their localization in the interstitial area. Spindle-shaped TCs were located, as a rule, between cardiomyocytes, and star-shaped—in free interstitial zones and around blood vessels.

Comparison of the results of a semi-quantitative TEM assessment of TCs presence and their morphological characteristics in atrial appendage of myocardium in patients with various forms of AF did not reveal any differences. However, when dividing patients by the presence of IAA, it was found that TCs score prevail in LAA of AF + IAA group than in AF group (Fig. 2e) (Mann–Whitney test, *p* < 0.05). In addition, in LAA myocardium it was significantly lower compared to RAA myocardium (Mann–Whitney test, *p* < 0.05) (Fig. 2f), and correlated with the duration of AF (r = 0.50; *p* = 0.0035) and with a high degree of mitral valve regurgitation (r = 0.37; *p* = 0.033) (Fig. 2g,h), which was often found in patients with AF. We found no correlation between the presence of TCs and interstitial fibrosis, hypertrophy of atrial appendages cardiomyocytes and myolysis in them.

**Figure 3:**
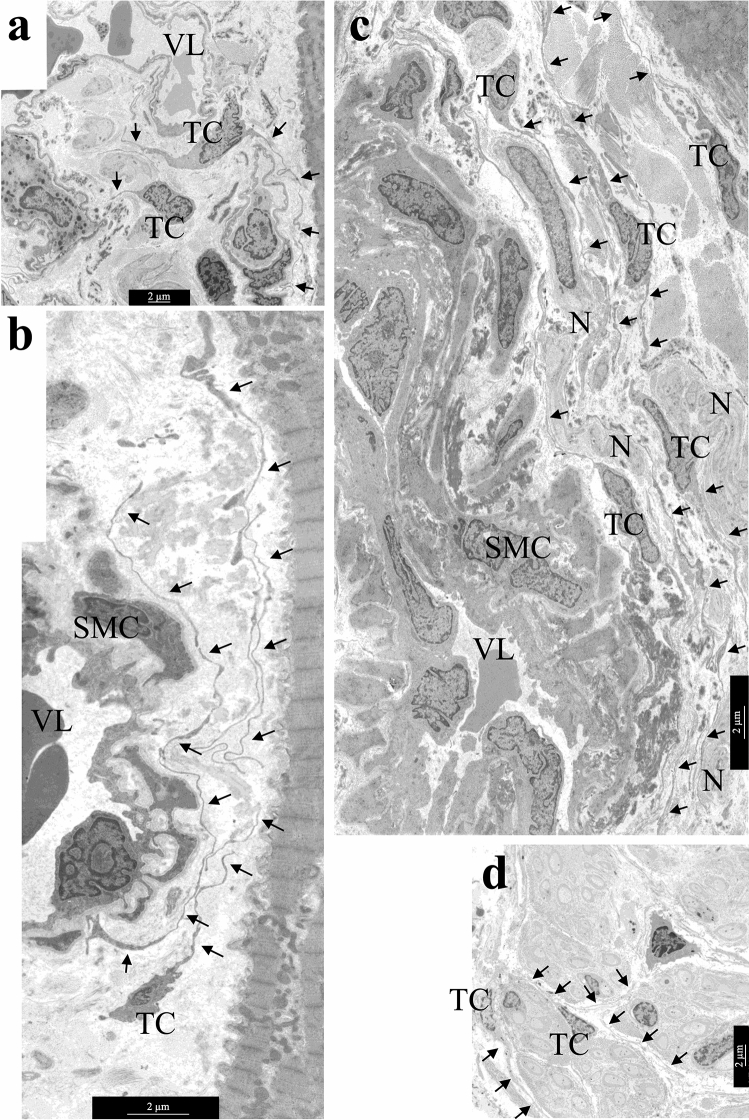
The location of TCs in the interstitial area—around large and small vessels, next to cardiomyocytes and nerve fibers. (**a**) TCs next to capillary. (**b**) TC near arteriole. (**c**) TCs in perivascular space**—**next to a relatively large vessel. (**d**) TCs inside the nerve fiber. TCs processes are indicated by arrows. TC—telocyte. N**—**nerve fibers. SMC—smooth muscle cells. VL—vessel lumen. bar 2 µm.

The TCs processes surrounded and “accompanied” the interstitial vessels of all types: they were located around the layer of capillary endotheliocytes, the layer of smooth muscle cells of small venules and arterioles, and were found in the perivascular zone next to large vessels (Fig. 3a–c). Atrial myocardium was characterized by the location of TCs in proximity to the nerve fibers—at a distance from them or even inside the nerve fiber (Fig. 3d).

**Figure 4:**
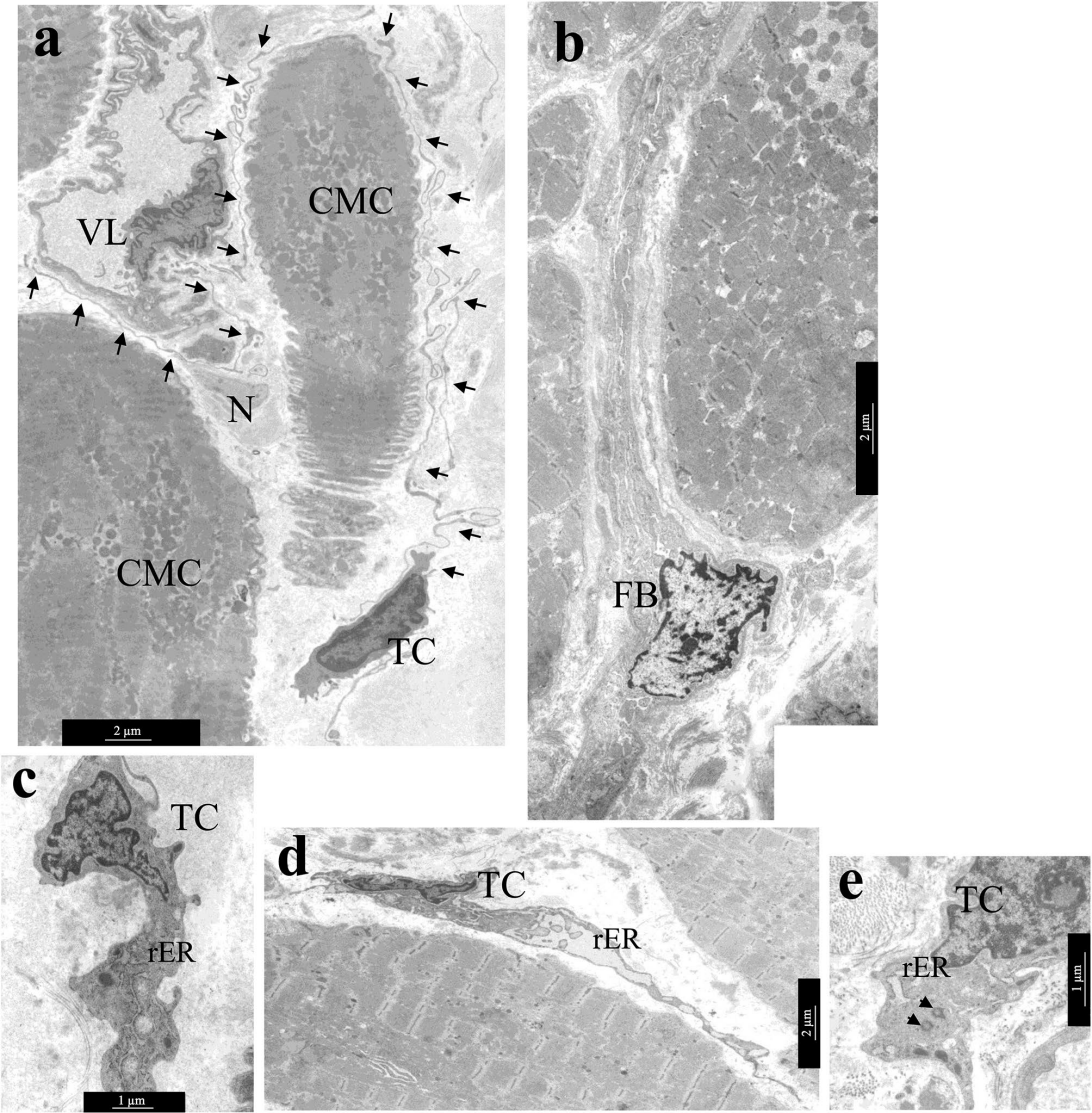
Ultrastructural features of atrial appendage TCs. (**a**,**b**) Comparison of ultrastructural morphology of TCs and fibroblasts of atrial appendage myocardium. (**a**) Features of TCs morphology: spindle-shaped cells with a small amount of cytoplasm in the perinuclear region, long thin processes (arrows) of TCs surround cardiomyocytes, blood vessels, nerve fibers. (**b**) Features of fibroblast (FB) morphology: star-shaped cells with a large volume of cytoplasm filled with GER tanks, thick processes of fibroblast in the interstitium. (**c**) rER tanks, mitochondria, and electron-dense inclusions in the perinuclear zone of TC cytoplasm. **(d**) In some TCs rER tanks are extended, filled with electron transparent contents. (**e**) Two centrioles in the cytoplasm of TC (arrows), rER tanks. (**a**,**b**,**e)—**bar 2 µm. (**c**,**d**)—bar 1 µm. TEM. TC—telocyte, FB—fibroblast. rER—rough endoplasmic reticulum. CMC—cardiomyocyte. N—nerve fibers. SMC—smooth muscle cells. VL—vessel lumen.

A comparative TEM analysis of the morphological features of TCs and fibroblasts demonstrated that despite the similarity of their localization in the interstitium, the ultrastructure of these cells differed greatly. The pathognomonic feature of TCs morphology was the presence of thin Tps with a diameter of 0.1–0.2 µm with local dilations. The diameter of TCs in the nucleus region was smaller than that of fibroblasts. Furthermore, significantly fewer rough reticulum (rER) tanks were found in the cytoplasm of TCs than in the cytoplasm of fibroblasts (Fig. 4a,b). TEM investigation revealed that the nuclei of TCs had an irregular shape with clumps of heterochromatin under the nucleolemma, they were surrounded by a small rim of cytoplasm containing single mitochondria, tanks of smooth and rER, the structures of the Golgi complex (Fig. 4c).

The ultrastructural features of TCs did not differ in patients with various forms of AF, however, small differences were found between the myocardial TCs of LAA and RAA appendages. In LAA myocardium of 82.1% patients and in RAA myocardium of 68% patients with AF, TCs contained a large number of rER in the cytoplasm, including dilated ones, which indicated a high secretory activity of TCs (Fig. 4d). In RAA myocardium, high secretory activity of TCs was detected in patients with prolonged episodes of cardiac arrhythmia (r = 0.51; *p* = 0.016) and high degree of tricuspid valve regurgitation (r = 0.52; *p* = 0.009). Besides, in both groups of patients with AF, TEM revealed centrioles in TCs (Fig. 4e): in LAA of 10.8% of patients and in RAA of 24% of patients, that confirmed the proliferative activity of these cells. The presence of centrioles in TCs of RAA positively correlated with the degree of fibrosis (r = 0.50; *p* = 0.043) and negatively with IAA (r = − 0.74; *p* = 0.037). In LAA, centrioles in TCs were detected in patients with a high degree of mitral valve regurgitation (r = 0.41; *p* = 0.032).

**Figure 5:**
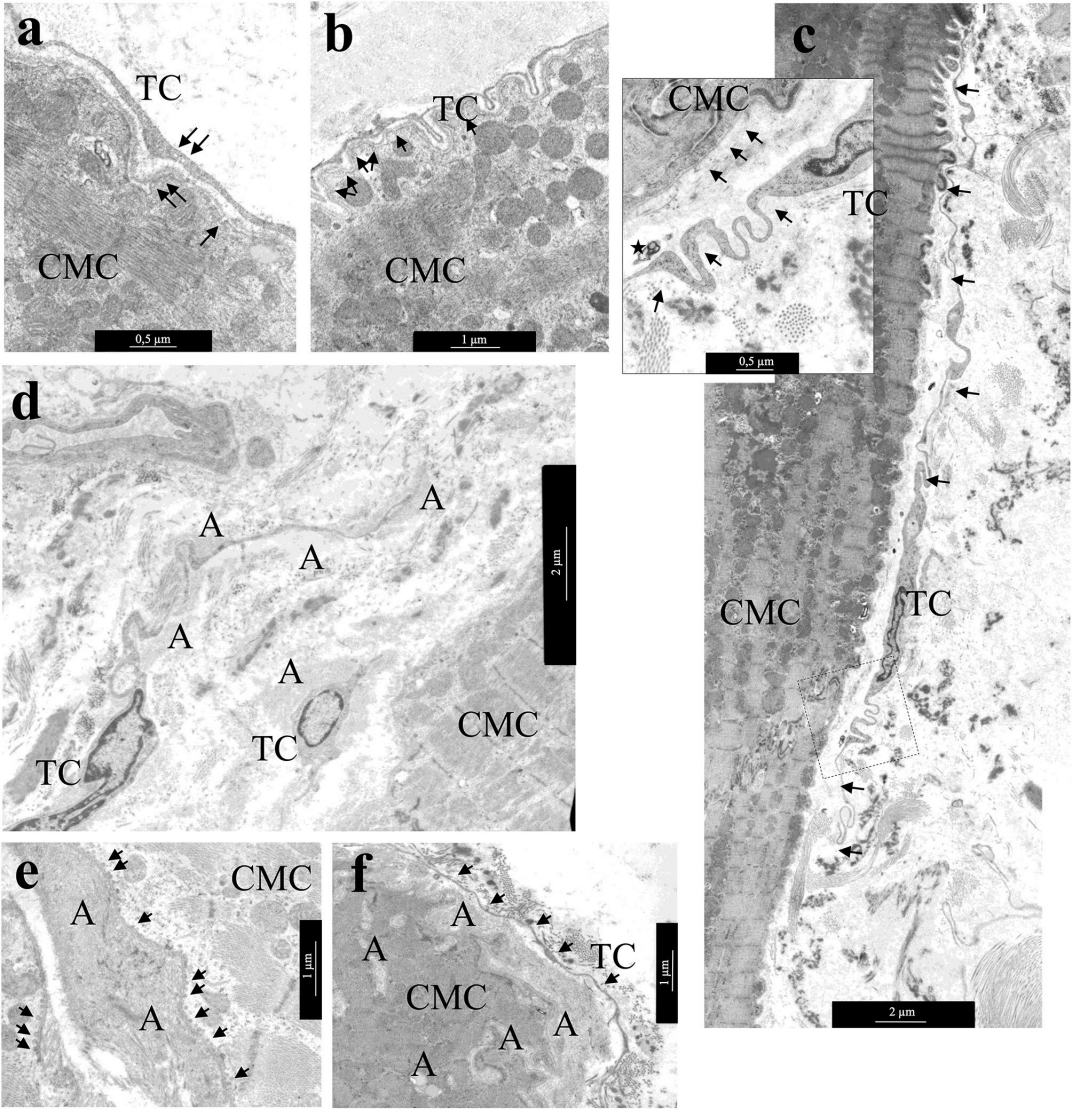
Distance interactions of TCs and cardiomyocytes. (**a**) The caveolae in the membrane of cardiomyocyte and TC process (arrows). bar 500 nm. (**b**) The process of TC follows all surface curves and is distributed in the invagination of the T-system of cardiomyocytes. Note the caveolae on the sarcolemma of a cardiomyocyte (arrows). bar 1 µm. (**c**) TC with extended Tps (arrows) located along the cardiomyocyte surface. bar 2 µm. Insert—Several caveolae on the surface of cardiomyocyte and TC (arrows). A multivesicular body (asterisk) in the interstitium next to the process of the TC. bar 500 nm. (**d–f**) TCs in areas of amyloidosis. (**d**) TCs and their processes are surrounded by amyloid fibrils. bar 2 µm. (**e**) Accumulation of amyloid fibrils on the surface of the cardiomyocyte membrane. Multiple vesicles (arrows) on the inner surface of the sarcolemma of the cardiomyocyte. (**f**) Amyloid fibrillar deposits on the surface of the cardiomyocyte membrane and in invaginations of the T-system of the cardiomyocyte. The TC process (arrows) at a distance from the cardiomyocyte membrane. (**e**,**f**)—bar 1 µm. TEM. TC—telocyte, CMC—cardiomyocyte, A—amyloid fibrils.

In atrial appendage myocardium of adult patients with AF, TCs rarely contacted with adjacent cells that they “accompanied”. Tps of the TCs were located, as a rule, at a distance of 0.3–0.9 μm from the surface of cardiomyocytes, followed all the curves of their sarcolemma (Fig. 5a,b). No direct contacts of TCs with stem cells and macrophages were also found. Although, they had been previously described in the myocardium of children with congenital heart defects^6,9,32^. At the same time, caveolae on opposite membranes of TCs and cardiomyocytes, exosomes, and multivesicular bodies located nearby in interstitium were probably related to the paracrine interactions between both types of cells (Fig. 5c).

In the areas of myocardial amyloidosis at the ultrastructural level, we revealed randomly organized amyloid fibrils with a diameter of 8–10 nm. Amyloid fibrils were located on the surface of the basal membranes of endotheliocytes and cardiomyocytes, they surrounded these cells and propagated in the invaginations of the T-system of cardiomyocytes. TCs with Tps were also surrounded by clusters of amyloid fibrils, which affected the effectiveness of their contacts with nearby cells (Fig. 5d). There were many vesicles on the inner surface of the cardiomyocyte sarcolemma opposite the zone of amyloid deposits on the outer surface of the sarcolemma (Fig. 5e). We suggested that intracellular vesicles contain ANP precursor which polymerized into the fibrillar form of amyloid in the interstitial area. The aggregation of amyloid fibrils on the surface of the plasmalemma of both TCs and cardiomyocytes as supposed could prevent their direct and paracrine contacts since Tps were located farther apart from the membrane of cardiomyocytes than usual (Fig. 5f).

**Figure 6:**
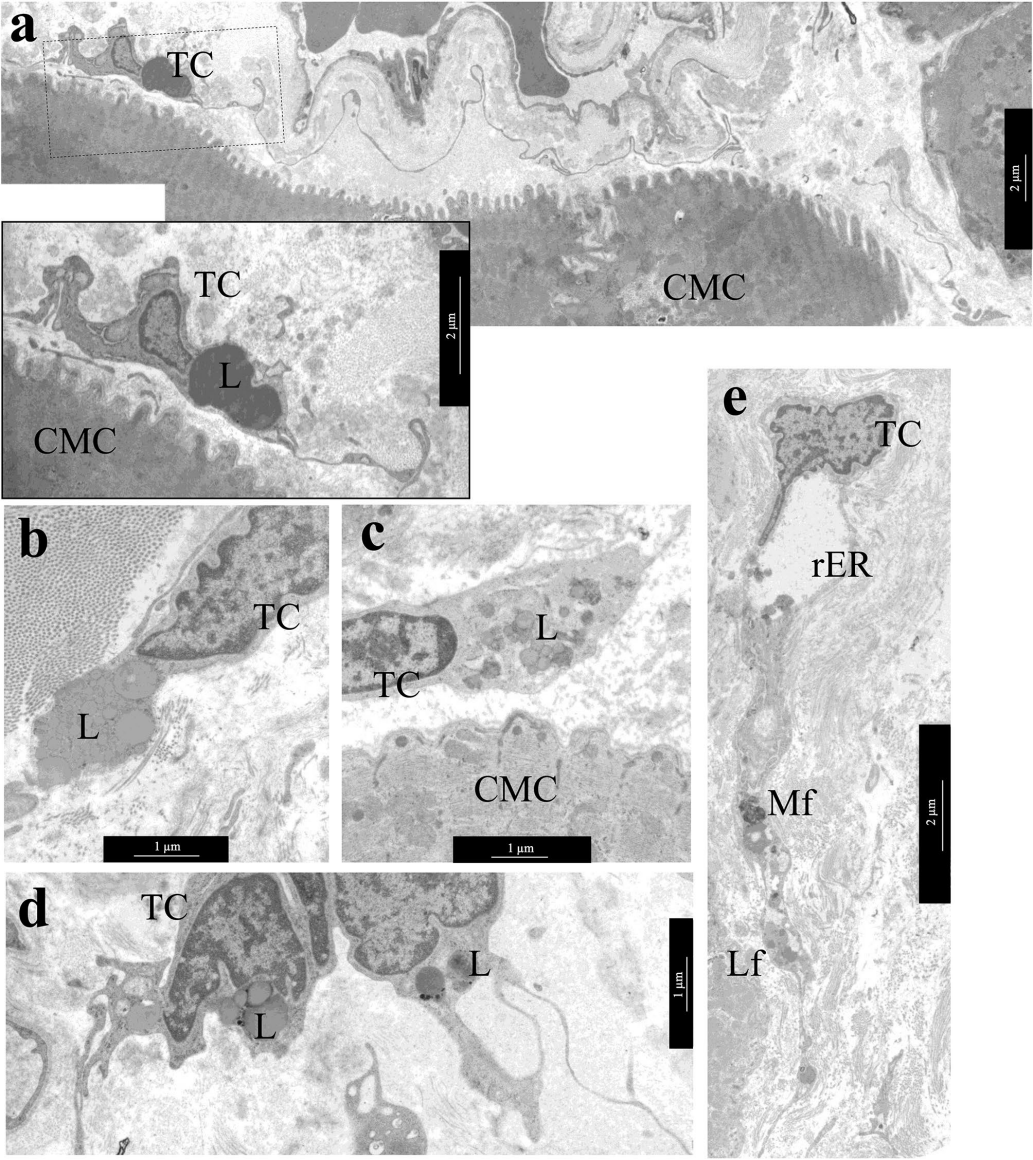
Lipid inclusions and dystrophic changes in atrial appendage myocardial TCs in patients with AF. (**a**) Single lipid inclusions in the perinuclear zone of the TC cytoplasm. bar 1 µm. Insert—bar 2 µm. **(b–d**) The accumulation of multiple small and large lipid inclusions in the perinuclear zone of the TCs cytoplasm. bar 1 µm. (**e**) Myelin-like figures (Mf), rough endoplasmic reticulum (rER) and lipofuscin granules (Lf) in the TC cytoplasm. bar 2 µm. TEM. TC—telocyte, CMC—cardiomyocyte. L—lipid inclusions.

An important ultrastructural feature of adult cardiac TCs was the accumulation of lipid inclusions in the cytoplasm of these cells in LAA 39.3% of patients and in RAA 52% of patients with different forms of AF, which was rare in children cardiac TCs^32^. Single and organized into clusters lipid inclusions of various sizes were found in the perinuclear zone of the TCs cytoplasm and, less commonly, in the dilations of Tps (Fig. 6a–d). In some TCs they filled the entire perinuclear zone of the cytoplasm. Single cardiac TCs contained small myelin-like figures and lipofuscin granules (Fig. 6e)**,** which were the signs of dystrophic changes in TCs of LAA of 14.3% of patients and RAA of 8% of patients. It should be noted that in atrial appendage myocardium of adult patients regardless of the form of AF hemodynamic overload provoked dystrophic changes in cardiomyocytes, one of the manifestations of which was the accumulation of lipid drops in their sarcoplasm. In TCs such changes, in our opinion, were a sign of the phagocytic activity of these interstitial cells. Thus, in AF, pathological changes in atrial appendage myocardium were complex and affected both the components of the stroma and parenchyma.

### Immunohistochemistry

**Figure 7:**
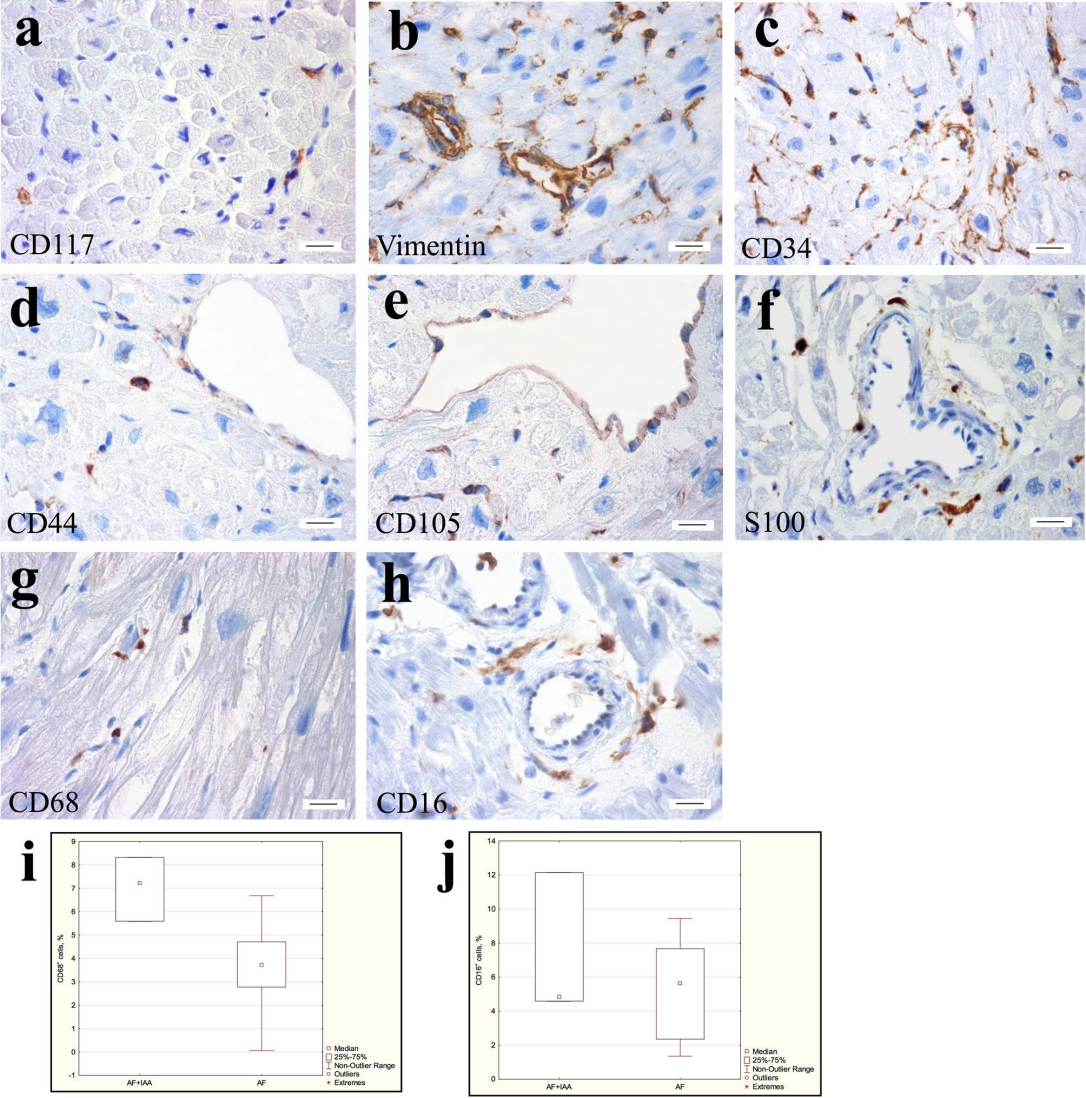
Immunohistochemical detection of TCs in atrial appendage myocardium of patients with AF. (**a**) Single CD117^+^ cells in the interstitium. (**b**) Vimentin^+^ TCs, endotheliocytes, and connective tissue cells. (**c**) CD34^+^ endotheliocytes, and TCs surrounding vessels, cardiomyocytes, and endotheliocytes in the interstitium. (**d**) CD44^+^ TCs in the interstitium, as well as weakly-labeled endotheliocytes. (**e**) CD105^+^ endotheliocytes. (**f**) S100-positively stained nerve fibers**. (g**) CD68^+^ round cells of histiocytic origin and spindle-shaped TCs in the interstitium. (**h**) CD16^+^ round cells of histiocytic origin and spindle-shaped TCs in the perivascular area. (**a–h**) Streptavidin–biotin complexes. bar 15 µm. (**i**) The number of CD68^+^ cells in the atrial appendage myocardium of the AF + IAA patients significantly exceeds the number of these cells in the AF group. Mann–Whitney test, *p* < 0.05*.* (**j**) The number of CD16^+^cells in the atrial appendage myocardium of the AF + IAA patients does not differ significantly from the number of these cells in the AF group. Mann–Whitney test, *p* > 0.05.

An immunohistochemical study revealed that atrial appendage myocardial TCs of patients AF expressed markers of stem cells (CD117^+^), fibroblast-like cells (vimentin^+^), hematopoietic stem cells (CD34^+^) and cell adhesion protein (CD44^+^), that mediated the adhesion of hematopoietic, fibroblast and glial cells with the extracellular matrix. No differences were found between the TCs immunophenotypes of patients with various forms of AF as well as between the AF and the AF + IAA groups. CD117, vimentin, CD34, and CD44 positively labeled TCs were small-sized, spindle- or star-shaped. They were located in the interstitium next to cardiomyocytes, small and large vessels (Fig. 7a–d). None of the described markers was specific for the TCs. Therefore, to identify TCs, we focused on the localization and structural features of these cells. Marker CD105 was found in RAA and LAA myocardial endothelial cells but was not detected in interstitial cells with morphology and localization similar to TCs (Fig. 7e). It should be noted that the marker of nerve cells S100 labeled cells that have similar localization to TCs (Fig. 7f). However, atrial myocardium contained a large number of nerve fibers, and we had confirmed this by studies at the ultrastructural level. Thus, since S100 was not a specific marker of TCs it should be carefully recommended for the identification of these cells.

Earlier in this study, we detected by TEM the lipid inclusions in the cytoplasm of atrial appendage TCs of patients from all groups with AF (Fig. 6a–d). We hypothesized that these ultrastructural changes were a consequence of the phagocytic activity of the TCs. To confirmed this by immunohistochemistry we used histiocytic cell markers CD68 and CD16. CD68 is a glycoprotein from the LAMP family, which is involved both in phagocytic activity and in intracellular lysosomal metabolism and is expressed on the surface of macrophages, lymphocytes, fibroblasts, and endothelial cells. CD16 is a transmembrane protein that is a receptor for the Fc fragment of IgG III, mediating phagocytosis. CD16 is expressed on macrophages, mast cells, neutrophils, eosinophils, and some T cells. We found CD16 and CD68 positively labeled cells in atrial appendages myocardium of patients from all groups with AF, both markers were expressed in a small number of round-shaped macrophages. Furthermore, both markers were expressed in spindle-shaped TCs located in the perivascular area of the interstitium and between cardiomyocytes: CD68 identified antigen-positive membrane-enclosed inclusions, while CD16 demonstrated a total membrane staining of such cells (Fig. 7g,h). Interestingly, in RAA myocardium of the AF + IAA patients the number of CD68-positive histiocytes and TCs-like cells with histiocyte signs was on average 7.04 ± 1.4% (median 7.22%) of cells and significantly exceeded the proportion of these cells in the AF group (4.5 ± 2.5%, median 4.2%) (Mann–Whitney test, *p* < 0.05) (Fig. 7i). The number of CD16-positive cells in the myocardium of the AF + IAA patients was 7.1 ± 4.3% (median 4.8%) and did not differ significantly from the AF group (5.9 ± 3.5%, median 5.9%) (Fig. 7j). Thus, immunohistochemical data supported the hypothesis of histiocytic activity of macrophages and TCs in atrial appendage myocardium of both groups of patients with predominant involvement of the AF + IAA patients.

## Discussion

TCs are a unique type of connective tissue cells. Thin long processes of TCs coordinate all cells of the cardiac interstitium and provide a connection between them, although TCs occupy only about 1.2 ± 0.3% of myocardial volume and 0.5–0.7% of interstitial tissue^2,9^. The present study has analyzed the ultrastructural and immunohistochemical features of TCs in atrial appendage myocardium of patients with various forms of AF and found no differences between them. TCs size allowed us to accurately identify them only at the ultrastructural level by the main morphological feature—the presence of long thin Tps surrounding cardiomyocytes, blood vessels, and nerve fibers. The diameter of TCs, according to our data, did not exceed 4 μm and the diameter of TCs processes was 0.1–1 μm. Tps were up to hundreds of microns in length with dichotomous branching and local dilations, they formed a branched network. Such a morphology features coincide with the literature for the myocardial TCs of human and another mammalian in vivo and in vitro^1–4,6–10,12,62^.

Atrial myocardium of patients with AF undergoes hemodynamic overload which leads to morphological changes in the interstitial tissue—an increase in the area of interstitial and perivascular fibrosis, disruption of tissue architectonics, and separation of interstitial cells^39–44^. Our morphometric study demonstrated in myocardium of patients with all forms of AF cardiomyocyte hypertrophy, with a partial loss of myofibrils (myolysis) in them followed by filling of free zones of sarcoplasm with glycogen granules and mitochondria, as described earlier^39^. In addition, patients with LSAF showed a significant increase in the length of the LAA cardiomyocytes compared to PAF and PsAF, which could explain the LA dilatation specific for AF.

In the present study, by analyzing Sirius red and immunohistochemistry-stained preparations, we detected ANP-positive amyloid deposits in the myocardium of both atrial appendages in 18.6–30.5% of patients with different forms of AF. ANP works as a precursor protein for amyloid deposition in IAA^41–43,48,56,57,59^. The accumulation of amyloid fibrils is thought to disrupt the formation of adequate intercellular contacts, electrical conjugation of neighboring cardiomyocytes and paracrine interactions of TCs with interstitial cells surrounding them. IAA serves as a morphological substrate for the development, promotion, and maintenance of AF. According to other authors, IAA has been found in atrial myocardium of 9.6–39% patients with valvular defects and persistent AF^42,58^ and reaches 60.89% of patients^43^. The inverse relationship is also true—in patients with IAA, AF has been revealed in 48.5–82.35% of cases^43,58^. According to our data, patients with AF + IAA were older and had a greater degree of mitral valve regurgitation than patients with AF (Table 2). A similar relationship of amyloidosis degree and the age of patients with AF has been shown previously^41,44,56,58^. For example, according to Krishnappa et al.^58^, in patients with AF and mitral valve disease older than 60 years, IAA is detected in 9.6% of cases, while in patients older than 80 years, their proportion increases to 20.4%. Apparently, the development and maintenance of AF is associated to a greater extent with age-related impairment in ANP processing and its accumulation in a fibrillar form in the interstitium. IAA is considered a predictor of the poor outcome of heart surgery. A 5-year retrospective analysis by Bhakhri et al.^50^ confirms an increase in the frequency of repeated episodes of dysrhythmia requiring implantation of a permanent pacemaker. Furthermore, the perioperative death rate in the group with IAA is elevated up to 11.5%, while in a group without dysrhythmia only 1.4%. The main problem in the diagnosis of IAA is the inability to verify this disease without examining the atrial myocardial biopsy.

**Table. 2.** Clinical characteristics of patients with different forms of AF (PAF, PsAF, LSAF) and two groups with AF and AF + IAA.

Clinical parameters	Patients with different forms of AF			Patients with IAA or AF + IAA	
	PAF (n = 12)	PsAF (n = 20)	LSAF (n = 23)	AF (n = 37)	AF + IAA (n = 18)
Age, mean ± SD, years	57.4 ± 8.6	57.5 ± 7.4	52.0 ± 10.7	53.1 ± 9.8	59.5 ± 7.1**
Male / Female	8 / 4	14 / 6	12 / 11	25 / 12	9 / 9
Left atrial volume, mean ± SD, ml	133.1 ± 26.3	127.8 ± 23.8	133.3 ± 25.4	131.3 ± 25.0	131.5 ± 24.7
Left atrial dimension, mean ± SD, mm	45.5 ± 5.0	48.7 ± 3.5	49.3 ± 7.1	48.2 ± 6.3	48.2 ± 4.2
LV ejection fraction, mean ± SD, %	65.3 ± 5.8	60.2 ± 6.7*	59.6 ± 8.0*	60.9 ± 6.7	61.4 ± 8.6
Duration of AF, mean ± SD, years (min–max)	10.0 ± 9.0 (1–27)	6.2 ± 5.6 (0.5–21)	6.0 ± 5.0 (1–20)	6.3 ± 5.9 (1–25)	8.3 ± 7.3 (0.5–27)
Arterial hypertension, % of patients	41.7	30	47.8	35.1	50
Mitral valve regurgitation, degree 2–3, % of patients	8.3	40	34.8	24.3	44.4**
Diameter of mitral valve fibrous ring, mean ± SD, mm	35 ± 3	34.6 ± 1.9	37.3 ± 3.7*†	35 ± 3	37 ± 4
Tricuspid valve regurgitation, degree 2–3, % of patients	0	15	26.1	10.8	22.2
Diameter of tricuspid valve fibrous ring, mean ± SD, mm	33.9 ± 2.8	35.3 ± 2.6	37.7 ± 4.0*	36 ± 3	37 ± 4

*Comparison with PAF, Mann–Whitney U Test *p* < 0.05.

^†^Comparison with PsAF, Mann–Whitney U Test *p* < 0.05.

**Comparison AF + IAA group with AF group, Mann–Whitney U Test *p* < 0.05.

Based on the analysis of clinical and morphological correlations obtained by us in the study of atrial appendages of the myocardium of patients with various forms of AF, we assumed that the structural remodeling of the myocardium in these cases was carried out differently. In PAF, cardiomyocyte hypertrophy and IAA negatively correlated with myofibril loss in cardiomyocytes, while mitral valve deficiency and LA dilatation positively correlated with myofibril loss in cardiomyocytes; atrial myocardial rearrangement occured as compensatory hypertrophy with an increase in the number of myofibrils in cardiomyocytes. In patients with PsAF, these associations were not revealed. In patients with LSAF, an increase in the LAA cardiomyocytes diameters correlated with an increase in LA size, dilatation of the mitral valve fibrous ring and with an increase in interstitial fibrosis in LAA myocardium. In LSAF, RAA cardiomyocytes hypertrophy and IAA were accompanied by loss of myofibrils in cardiomyocytes. The remodeling of the atrial myocardium in LSAF, with a decrease in the number of myofibrils and an increase in cardiomyocyte length, was similar to ventricular myocardial hypertrophy at the decompensation stage.

TEM investigation shown the presence of TCs in the interstitium of both atrial appendage myocardium of patients with different forms of AF. In addition, in the AF + IAA group the TCs score in LAA myocardium was significantly higher than in patients in the AF group and positively correlated with the duration of AF and a high degree of mitral valve regurgitation. There were a large number of rER cisterns in the cytoplasm of TCs, which indicated a high synthetic activity of these cells. The presence of rER in the cytoplasm of LAA TCs correlated with the degree of IAA, while in RAA TCs—interrelated with dysrhythmia episodes duration and tricuspid valve regurgitation. TCs are supposed to be additional sources of excitation and/or ways of conducting an electric impulse in atrial myocardium, they play the role of pacemaker cells and serve as an intermediary between the vessels, cardiomyocytes and nerve fibers^1,2^. In the myocardial TCs of both atria appendages of patients with AF, we also found centrioles—organelles involved in the organization of the spindle of division and necessary for cell proliferation. The presence of centrioles in RAA TCs positively correlated with the degree of fibrosis and negatively with IAA, in LAA TCs it was associated with a high degree of mitral valve regurgitation. It is evident, that synthetic activity and proliferation of atrial appendage TCs were activated in the hemodynamic overload myocardium.

The long thin processes of the TCs (Tps) “accompany” all the components of the interstitium—cardiomyocytes, vessels of all types, nerve fibers. Interestingly, according to our observations, in atrial appendage myocardium of patients with AF, Tps were always located at some distance from other cells, suggesting paracrine regulation of their activity. The distance from Tps to the surface of cardiomyocytes and nerve fibers reached 0.9 μm, which slightly exceeded the data of other authors^1,2^. Perhaps this is a consequence of remodeling of the myocardial interstitium with an amyloid deposition that leads to poor contact of TCs with surrounding cells. We also were not able to detect direct contacts between TCs and cardiomyocytes, but observed caveolae on the membranes of oppositely located cells and multivesicular bodies in the interstitium next to the Tps. It is believed that through multivesicular bodies, TCs carry out paracrine interactions with adjacent cells. In recent years, TCs are known to produce interleukins IL-6, IL-2, IL-10, IL-13, VEGF, macrophage inflammatory proteins 1a (MIP-1a), MIP-2 and MCP-1^63^, angiogenic microRNAs^38^. For example, through exosomal miRNA-21-5p, cardiac TCs ensure the survival of microvascular endothelial cells in myocardial infarction^64^.

TCs act as immunoregulators expressing toll-like receptors TLR 2, TLR 3, TLR 5^65^. Transfer of TCs exosomes into infarct-affected rat myocardium attenuates cardiac fibrosis, improves cardiac function, and increases angiogenesis^36^. Similarly, in vitro cultivation of endotheliocytes in the presence of exosomes isolated from rat myocardial TCs stimulates proliferation, migration, and the formation of capillary-like structures by endotheliocytes^36^.

In atrial appendage myocardium of adult patients with AF, we did not found contacts of TCs with myocardial stem cells, previously described in children with congenital heart defects^2,6,9,31,32^. Some authors suggest that in children's myocardium, TCs are a component of the stem cell niche, provide their maturation, directed migration to the sites of differentiation. TCs take part in the formation of correct three-dimensional pattern of myocardial tissue^1,2,31,37^. In adult myocardium of patients with AF, TCs seem to perform other functions and their interaction with stem cells is impaired or not carried out.

In this work, the ultrastructural changes of the atrial appendages myocardial TCs of adult patients with AF and AF + IAA were described in detail. We revealed, for the first time, lipid inclusions in the cytoplasm of some TCs, which formed conglomerates and sometimes filled almost the entire perinuclear zone. In other cases, single lipid drops were found in the perinuclear zone and dilations of Tps. Such changes in TCs ultrastructure corresponded to changes in their functions. Lipid inclusions interfered with the intracellular transport of substances in the cytoplasm, disrupted the synthetic function, and led to their inferiority. An analysis of the literature data regarding the ultrastructural features of TCs in the myocardium of patients with cardiovascular pathology has not found similar references. Some authors note the compression and shortening of Tps and the appearance of vacuoles in the cytoplasm (without identifying the cause of these changes), while the accumulation of lipid drops have not been described^60^. One of the most probable causes of lipid accumulation in the cytoplasm of TCs we believe activation of phagocytic activity of these cells, the possibility of which is confirmed in studies of CD34-positive TCs of the colon and skin^66^. We assume that remodeling of atrial myocardium with an increase in fibrosis and accumulation of amyloid fibrils in the interstitium progresses in adult patients with AF and leads to dystrophic changes in cardiomyocytes, their destruction, and, as a result, enhanced phagocytic activity in interstitial cells, including TCs.

There is no reliable data on the origin of either telocytes in the literature. It can be assumed that TCs are pro-epicardial origin and of a mesenchymal nature, like other epicardial cells, undergo epithelial-mesenchymal transformation^67^, migrate from myocardial epicardia, where they acquire the properties of interstitial perivasular fibroblast-like cells. It is important to consider the differences between TCs and fibroblasts, the main cellular components of the interstitium. At the ultrastructural level, the difference in morphology and hence the function of these cells is obvious. Fibroblasts, unlike TCs, have thick short processes without dilations, a significantly large volume of cytoplasm, in which rER tanks predominate. Fibroblasts rarely contain caveolae and lack a basement membrane. They are often surrounded by collagen fibers. The main function of fibroblasts is the production of extracellular matrix components and control for the synthesis and degradation of its components. While TCs, having a branched network of Tps, apparently perform signaling functions and regulate the activity of all cellular components of the interstitium^1,2,9,13^. TCs differ from fibroblasts in the expression patterns of several genes^25,68,69^ and when cultured in vitro TCs are characterized by a higher ability for adherence, spreading, and Tps extension than fibroblasts^70^. In the present work, we were not able to register contacts between TCs and fibroblasts or to find a reliable relationship between TCs presence and the degree of fibrosis. Nevertheless, we noticed that the number of TCs was reduced in areas of fibrosis, which was probably due to their transformation into fibroblast-like cells. Similarly, Mitrofanova et al.^59^ indicates that the number of CD34^+^/S100^+^ TCs inversely correlates with the area of fibrosis and lipomatosis in the myocardium of patients with long-term persistent AF. In patients with systemic sclerosis, the content of CD34^+^/CD31^-^ TCs in the left ventricular myocardium and lungs is significantly reduced compared with control samples^27^. In the myocardium of patients with end-stage heart failure caused by idiopathic dilated cardiomyopathy, ischemic and inflammatory cardiomyopathy and aortic valve stenosis the number of C-kit^+^ TCs/mm^2^ is also decreased more than twofold compared to the intact myocardium and inversely correlates with the amount of mature fibrillar collagen type I and collagen propeptides PINP and PIIINP^60^. In the zone of experimentally induced rat infarction, CD34^+^ and C-kit^+^ TCs are either not determined^61^ or their number is significantly reduced^7,33^.

In this work, in atrial appendages myocardium of patients with AF by immunohistochemistry we revealed interstitial cells, which, taking into account their location and morphological features could be attributed to TCs. These cells corresponded to immunohistochemical signs of stem cells (CD117^+^), fibroblast-like cells (vimentin^+^), endotheliocytes (CD34^+^), fibroblast, and glial cells (CD44^+^). While marker of cells with neurogenic differentiation (S100^+^) was observed in nerve fibers, and the marker of activated endotheliocytes (endoglobin^+^/ CD105^+^) was found only in endotheliocytes, but not in TCs. Immunohistochemical markers listed above have been described in the literature and generally accepted for the detection of myocardial TCs^2,7,12,17,33,36,60,71^. Since none of these markers is specific, a number of authors suggests using double immunohistochemical staining for identification of TCs: CD34^+^/CD31^-^, in contrast to CD34^+^/CD31^+^ endothelial cells^27,29^, CD34^+^/PDGFR-α^+^^10^, CD34^+^/S100^+^^59^, as well as C-kit^+^/vimentin^+^^36^. The literature actively discusses the possibility of inaccurate identification of TCs at the light-optical level using different markers of IHC staining, in particular, during the differentiation of epicardial TCs, among which there may be cells expressing markers of lymphatic endothelial cells^72^. Thus, the absence of specific markers of TCs forces researchers to use various markers to identify these cells and, in our opinion, indicates the heterogeneity of their population or their ability to differentiate into other cell lines.

The results obtained by us and discussed by other authors indicate that TCs are a highly plastic cell population. TCs properties and immunophenotype vary depending on the type of organ and the functional state of its interstitial tissue^73^. We cannot exclude the possibility of TCs differentiation with the acquisition of immunomorphological features of fibroblast-like cells and endotheliocytes. At the same time, we first have demonstrated the expression of markers of histiocytic differentiation (CD68 and CD16) by TCs in adult atrial appendage myocardium. We have shown that CD68-positive histiocytes and TCs are more common in the myocardium of patients in the AF + IAA group than in the AF group. Probably, during myocardial remodeling in the AF + IAA not only the number of histiocytes but also the number of TCs-like “transitional cells” with histiocytic function increases in the interstitium. This is, in our opinion, a manifestation of compensatory mechanisms, which are expressed by a change in the structure and functions of these interstitial cells during arrhythmogenic myocardial remodeling complicated by age-dependent IAA. The mechanism of transition of one cell type to another type of cells through "transitional" forms has been demonstrated in the cell culture^74^. Apparently, this is not well understood fundamental biological phenomenon that provides compensatory and adaptive processes in tissue and organ. However, in order to confirm this, an integrated approach should be used, including the analysis of ultrastructural features of cells.

Currently in the human myocardium by the method of single-cell and single-nucleus RNA sequencing, in accordance with the analysis of myocardial cells transcriptome, the following 11 major cell types were identified: atrial and ventricular cardiomyocytes, fibroblasts, endothelial cells, pericytes, smooth muscle cells, immune cells (myeloid and lymphoid), adipocytes, mesothelial cells and neuronal cells^75^. Other authors in the human myocardium identified more than 20 cell subtypes, which differed significantly by cardiac chamber, laterality, and gender^76^. In the above studies, there is no mention of TCs and it seems difficult to determine to which type of cells they can be attributed, since TCs simultaneously express markers of different types of cells**—**fibroblast-like, endotheliocytes, and, as we have shown, histiocyte-like. In addition, it can be assumed that the cell transcriptome may change depending on the microenvironment and composition of the extracellular matrix. We believe that none of the methods for studying isolated cells or their nuclei, including transcriptome analysis, can provide a complete picture. In this regard, morphological and especially ultrastructural identification of cells is very important. Popescu et al.^77^ suggested using the electron microscopy as a "platinum" standard for identification of TCs, including the analysis of the cell membrane, the presence of caveolae, mitochondria, endoplasmic reticulum, the number and thickness of processes, their location, and the presence of contacts with other cells.

The phenotype of CD68^+^, CD16^+^ transitional forms of TCs we found in the atrial appendage myocardium of patients with AF is similar to the previously described morphology of cardiac resident macrophages (so-called M2 macrophages)^78,79^. According to Hulsmans et al.^79^, these spindle-shaped, CD68^+^ and CD163^+^ resident macrophages with long processes that contact each other and with stromal cells are much more numerous in the human AV node than in the working myocardium, they express genes involved in electrical conductivity. In addition, M2 macrophages are involved in the regeneration of the heart when it is damaged, they are necessary for the proliferation of cardiomyocytes and are involved in angiogenesis^78,80^. The method of single nuclear RNA-sequencing of the transcriptome of human myocardial cells has shown that tissue-resident cardiac M2 macrophages associated with cardiovascular remodeling are characterized by expression of the lymphatic endothelial hyaluronan receptor LYVE1, scavenger receptors CD163 and COLEC12, the mannose receptor ubCH1, the E3 and liquin natural resistance-associated macrophage protein 1 (NRAMP1 or SLC11A1). Further isolation of subclusters revealed two more populations of cells that both express M2-polarization associated genes, including RBPJ and F13A1 and the transmembrane collagen COL23A1^75,76^. Hulsmans et al.^79^ do not exclude the possibility of the participation of M2 macrophages in conduction abnormalities not only in the AV node, but beyond the AV node, including AF. However, what cells did the authors^79^ describe in his work**—**macrophages or TCs**—**is still a subject of debate. Given the complexity of the classification and accurate identification of these cells in the myocardium, we assume that the TCs described in our work are a “transitional form” of TCs with signs of resident macrophages. It is possible that the presence of a multiple immunohistochemical phenotype in TCs does not indicate a complete change in the direction of their differentiation, but reflects only the transition from one stable state of differentiation to another in the pathologically altered atrial myocardium in AF. A similar concept is discussed in the article^81^, where instead of the state of terminal differentiation of cells, it is proposed to consider the state of stable differentiation. We hypothesize that this adaptive mechanism may be activated in the interstitium of the remodeled atrial myocardium in aged AF + IAA patients with hemodynamic overload and AF.

We have proposed the following model describing the role of TCs in a pathologically altered myocardium in patients with AF. Hemodynamic overload of the left ventricle due to mitral valve defect leads to remodeling of the left atrial myocardium, the development of fibrosis, lipomatosis, an increase in the size of the atrium and includes a compensatory mechanism associated with the active production of ANP by cardiomyocytes. In patients with IAA, excessive production of ANP leads to its deposition in the interstitial area in the fibrillar form. Clusters of amyloid fibrils surround cardiomyocytes and all interstitial components, including TCs, isolate them and disrupt intercellular contacts. Under these conditions, there is an increase in phagocytic activity with an activation of synthetic function and proliferation of the TCs. Such compensatory changes in the structure and function of TCs are more pronounced in the myocardium of patients with impairment of valves function, long duration of AF. Changes in the morphology and, accordingly, TCs function are not the cause, but either the consequence of adaptation of the atrial appendage myocardium to function in AF + IAA states or part of the general age-related myocardial remodeling in AF + IAA. Disorganization of the regulatory function of TCs, along with IAA, probably provokes inadequate electrical impulse conduction and promote AF patients. Taken together, our results provide novel insight into cardiac TCs function, suggesting the universal mechanism for the development and maintenance of AF in the atrium with age-dependent IAA which is based on the disruption of contacts between cardiomyocytes and stromal cells, one of the most important components of which are TCs and the transformation of TCs with the acquisition of the properties of histiocyte-like cells.

### Conclusion

The present study confirms an increase of interstitial and perivascular fibrosis and lipomatosis and age-related development of IAA in atrial appendage myocardium of patients with PAF, PsAF, LSAF. IAA was an age-related factor of the AF myocardial remodeling and was detected in 4.3–25% biopsies of LAA and 21.7–41.7% biopsies of RAA myocardium. TCs were identified in atrial appendages myocardium of patients with all forms of AF, but more often in the AF + IAA group than in the AF group. In LAA myocardium, TCs presence correlated with the duration of AF, the degree of age-depended IAA, and also with a high degree of mitral valve regurgitation. However, there was no correlation between the TCs presence and the degree of fibrosis and cardiomyocyte hypertrophy. Ultrastructural signs of the phagocytic activity of myocardial TCs of adult patients in combination with the synthetic and proliferative activity were described for the first time. Adult atrial appendage myocardial TCs corresponded to CD117^+^, vimentin^+^, CD34^+^, CD44^+^, S100^-^, CD105^-^ immunophenotype. No differences in morphology and immunophenotype of myocardial TCs were found in patients with various forms of AF. Immunohistochemistry also demonstrated the phagocytic activity of TCs-like cells in atrial appendage myocardium (CD68^+^, CD16^+^ labeling). CD68-positive interstitial cells were detected predominantly in the AF + IAA group than in the AF group. We suppose that in aged AF + IAA patients hemodynamic overload and pathological remodeling of atrial myocardium initiates the transformation of TCs, with the appearance of “transitional forms” combining morphological and immunohistochemical signs of fibroblast-like cells, histiocyte-like cells, and endotheliocytes. All of the above allows us to speculate about the undisclosed mechanisms of cellular plasticity, which accompanies the processes of age-related and pathological compensation and adaptation of organs and tissues.

#### Study limitations

This study has limitations associated with estimating the number of TCs since the most accurate identification of these cells is possible only at the ultrastructural level. It is not possible to take a large biopsy sample without the risk of harming the patient during surgery. Tissue processing requirements for TEM also limit the size of the biopsy. Another significant limitation of studies on human myocardium by TEM is the lack of comparative studies in the intact heart associated with the requirements for immediate tissue fixation to avoid autolysis. Therefore, we had to use the atrial appendage myocardium of patients with AF as a comparison group for the patients with AF + IAA.

Additional researches will be needed to expose the role of TCs in atrial myocardial remodeling in patients with AF. Moreover, further TEM investigations are required to determine the function of TCs and the directions of their differentiation in pathologically altered myocardium of patients suffering from cardiovascular diseases.

## Methods

Human cardiac myocardial samples from left atrial appendage (LAA) and right atrial appendage (RAA) were collected from 55 adult patients (aged 24–71 years) with AF, documented by electrocardiographic monitoring (12 patients with paroxysmal AF (PAF), 20 patients with persistent AF (PsAF), 23 patients with long-standing persistent AF (LSAF). The duration of the arrhythmic history was from 6 months to 27 years. All patients underwent the modified radiofrequency maze procedure («Labyrinth 3B») between May 2016 and December 2017 at A.N. Bakulev National Medical Research Center of Cardiovascular Surgery, the Ministry of Health of Russian Federation, Moscow, Russia. Intraoperative biopsies were taken as part of the research process. All patients before operation were evaluated by transthoracic echocardiography and computed tomography of the left atrium. Table 2 summarizes the clinical characteristics of the study subjects. Patients with LSAF and PsAF showed a significant decrease in LV EF compared to the PAF group. Mitral and tricuspid valve fibrous rings dilatation was noted in LSAF patients, compared to PAF and PsAF patients. (Mann–Whitney, *p* < 0.05). No patients with diabetes were found. 30–47.8% of patients with different forms of AF have suffered from arterial hypertension (2nd–3rd stage). Previously, only 4 patients (one from the PAF group and three from the PsAF group) underwent radiofrequency ablation of pulmonary vein.

According to the results of histological examination of the atrial appendage myocardial biopsy at light microscopy (Sirius red stain, immunohistochemical detection of ANP) and TEM (determination of deposits of amyloid fibrils), patients were divided into 2 groups: patients with AF and signs of IAA (AF + IAA group, n = 18) and patients with AF without signs of IAA (AF group, n = 37) (Table 2). The age of patients in the AF + IAA group averaged 59.5 ± 7.1 years (40–71 years), which was significantly older than in the AF group**—**53.1 ± 9.8 (24–66 years) (Mann–Whitney, *p* < 0.05). AF + IAA patients showed a high degree of mitral valve regurgitation and were more likely to suffer from arterial hypertension, than patients in the AF group (Mann–Whitney, *p* < 0.05). The AF + IAA group included patients with all forms of AF, presented in almost equal proportions (PAF**—**28%, PsAF**—**39%, and LSAF**—**33%), while the AF group predominantly consisted of patients with LSAF (52%) and PsAF (33%) forms of AF.

### Ethics approval

The study was performed according to the World Medical Association Declaration of Helsinki and approved by the Ethical Committee of A.N. Bakulev National Medical Research Center of Cardiovascular Surgery (record №1 29.04.2016). Investigation of the patients' biological materials was legally confirmed by the patients' informed consent.

### Tissue block preparation: light microscopy

Myocardial fragments were fixed in 4% buffered formalin, processed by a standard technique, and paraffin-embedded sections (3–4 μm thick) were stained with hematoxylin and eosin, Masson’s trichrome (Bio-Optica), Sirius Red (Bio-Optica) and used for histological examination. Masson’s Trichrome-stained sections were used in total for morphometric evaluation of percent fibrosis at × 100 using the Image-Pro Plus program (version 6.0.0.260; Media Cybernetics, USA). Sirius red-stained sections were used for a semi-quantitative 5-point assessment of the amyloid deposits distribution in the interstitium around blood vessels and cardiomyocytes: 0**—**no deposits; 1**—**deposits were around single cardiomyocytes; 2**—**around small groups of cardiomyocytes; 3**—**around half of cardiomyocytes and single vessels; 4**—**around more than 75% of cardiomyocytes and blood vessels. The slides were examined by light microscope Leica DMRB with Leica DFC495 camera and Leica PL FLUOTAR 10 × /0.3 and Leica N PLAN 40 × /0.65 (Leica Microsystems Gmbh, Austria).

### Thin-section light microscopy and transmission electron microscopy (TEM)

Atrial appendage biopsies collected from all patients were also processed for thin-section light microscopy and TEM. In brief, small fragments of the myocardium were pre-fixed in 2.5% glutaraldehyde and 1% paraformaldehyde in 0.1 M phosphate buffer (pH 7.4), post-fixed in 1.5% osmium tetroxide solution, dehydrated in graded concentrations of alcohol and embedded in Araldite. Semithin sections (1 μm thick) were stained by the Periodic acid–Schiff (PAS) method with additional methylene blue staining. The diameters of cardiomyocyte were measured passing through the nucleus in at least 50 cells for each patient (semithin sections, × 1000). In the same cardiomyocytes, the degree of myofibrils loss (myolysis) was determined on a 4-point scale**—**the zones without myofibrils occupied 10% (0), 10–50% (1), 50% (2) and more than 50% (3) of sarcoplasm.

Ultrathin sections were cut using ultramicrotome Leica ultracut UCT (Leica Microsystems Gmbh, Austria). Ultra-thin sections (50–70 nm) were collected on copper grids, contrasted with uranyl acetate and lead citrate, and examined using a Philips CM100 transmission electron microscope (Philips/FEI Corporation, Eindhoven, Holland). To assess the presence of the TCs at the ultrastructural level (× 4800), a semi-quantitative 6-point scale was used: TCs and their processes were absent (0); there was at least one TC in one field of view (1); several TCs and their processes were in less than half of the fields of view (2); in half the fields of view (3); in most fields of view (4); in all fields of view (5). TCs and prolongation diameters were measured at ultramicrophotographs (× 4800).

### Immunohistochemistry

For the immunohistochemical study, formalin-fixed paraffin-embedded tissue sections (3–4 μm thick) were mounted on adhesion slides (Thermo Scientific Menzel-Gläser, Germany). Deparaffinized and dehydrated sections were rinsed in phosphate buffer solution (PBS) at pH 7.4, incubated in hydrogen peroxidase block solution at 37 °C for 15 min, washed twice in PBS. Then 0.01 M citrate retrieval solution (pH 6.0) at 90 °C for 20 min were used. After that sections were cooled to room temperature for 20 min, washed twice in PBS and incubated with primary antibodies,as follows: CD117/C-kit rabbit, monoclonal,ready-to-use (clon YR145, Cell Marque, USA), vimentin rabbit, monoclonal, ready-to-use (clon SP20, Spring Bioscience, USA), CD34 mouse, monoclonal, ready to use (QBEnd/10; Spring bioscience, USA), CD44 mouse, monoclonal, 1:50 (Cell Marque, USA), CD68 rabbit, monoclonal,ready-to-use (clon SP251, Spring Bioscience, USA), CD16 rabbit, monoclonal, 1:100 (clon SP175, Cell Marque, USA). Then sections were treated with secondary anti-mouse and anti-rabbit antibodies with streptavidin–biotin complexes (SBK KIT DAKO, Denmark) following standard manufacturer's instructions. After that sections were washed twice in PBS and counter-stained with hematoxylin. The positive reaction products were detected by brown staining of the cells. Negative control was obtained by omitting the primary antibodies in the standard immunohistochemical protocol. The slides were examined by light microscope Leica DMRB with Leica PL APO 63 × /1.4 oil. Representative photomicrographs were taken using and Leica DFC495 camera (Leica Microsystems Gmbh, Austria). The numbers of CD68 and CD16 immunopositive cells in the interstitium were counted (× 400) over the whole histological sections and presented as a fraction of the total number of cells.

### Double immunofluorescence

For ANP/desmin double immunofluorescence reaction paraffin-embedded tissue sections were deparaffinized and dehydrated. Then sections were treated with 0.01 M citrate retrieval solution (pH 6.0) at 90 °C for 20 min, cooled to room temperature, washed twice in PBS and incubated with 5% bovine serum albumin (SAFC A3059) and 0.5% Triton X-100 (Sigma-Aldrich, T9284) in PBS for 60 min to block non-specific binding sites. Then sections rinsed in PBS and incubated with a cocktail (1:1) of primary antibodies for 90 min at 37 °C: antibody to the Atrial Natriuretic Peptide, mouse monoclonal,1:300 (Millipore, CBL 66, clone 23/1) and desmin, rabbit monoclonal 1:500 (ab32362, Abcam, UK). After washing in PBS sections were incubated with a cocktail (1:1) of two secondary antibodies conjugated with fluorochromes: Alexa 488, goat anti-rabbit 1:300 (A11008, Invitrogen Molecular Probes, USA) and Alexa 546, goat anti-mouse 1:300 (A11003, Invitrogen Molecular Probes, USA). The nuclei were stained with 4′, 6-diamidino-2-phenylindole (DAPI, Sigma-Aldrich, Germany). Immunofluorescence-stained preparations were embedded in Immu-mount (Thermo-Shandon) and examined using confocal laser microscope Leica TCS SPE (Leica Microsystems Gmbh, Austria).

### Statistical analysis

Statistical analysis was performed using the Statistica 10.0 software program package. The corresponding clinical data of patients and myocardial morphometry data obtained through research myocardium of patients with different forms of AF as well as between AF and AF + IAA groups were compared using the non-parametric Mann–Whitney test. Differences between groups were considered significant at *p* < 0.05. The associations between TCs presence, their ultrastructural and immunohistochemical features and clinical and morphological parameters of the myocardium of patients with AF were determined using the non-parametric Spearman correlation coefficient at *p* < 0.05 (r and p values were given in text).

## Abbreviations

AF: Atrial fibrillation
PAF: Paroxysmal atrial fibrillation
PsAF: Persistent atrial fibrillation
LSAF: Long-standing persistent atrial fibrillation
LAA: Left atrial appendage
RAA: Right atrial appendage
TCs: Telocytes
Tps: Telopodies
IAA: Isolated atrial amyloidosis
ANP: Atrial natriuretic peptide
TEM: Transmission electron microscopy
